# Current status of autologous stem cell transplantation for multiple myeloma

**DOI:** 10.1038/s41408-019-0205-9

**Published:** 2019-04-08

**Authors:** Rama Al Hamed, Abdul Hamid Bazarbachi, Florent Malard, Jean-Luc Harousseau, Mohamad Mohty

**Affiliations:** 1 Service d’hématologie clinique et thérapie cellulaire, Hôpital Saint-Antoine, INSERM UMRs 938 and université Sorbonne, Paris, France; 2 Institut de Cancerologie de l′Ouest, Centre René Gauducheau, Nantes-St Herblain, France

## Abstract

More than 30 years after its introduction, autologous stem cell transplantation (ASCT) remains the standard of care for young patients with newly diagnosed multiple myeloma. Not only did the arrival of novel agents such as immunomodulatory drugs (IMiDs), proteasome inhibitors (PI) and monoclonal antibodies not replace ASCT, instead they solidified its central role as standard of care. Novel agent use is now inarguably essential in induction, maintenance, and possibly consolidation. In light of these new advancements, new challenges arise in deciding on optimal practice. Who is most suited to undergo ASCT? Is there an age threshold that should not be surpassed? Should transplantation be embarked on early or is it reasonable to delay it? What are the optimal induction, consolidation, and maintenance therapies? What is the role of tandem transplantation in the era of novel agents and where do patient-specific cytogenetics come into the equation when deciding on treatment? These are some of the questions addressed in this review which we will attempt to answer with the latest currently available data.

## Introduction

Multiple myeloma accounts for approximately 10% of hematologic cancers and 1% of all cancers in general^1^. Thirty years after the work of Powles, Barlogie, and McElwain led to the initiation of the concept of high-dose therapy (HDT) followed by autologous stem cell transplantation (ASCT)^2–4^, transplantation remains the standard for treating newly diagnosed multiple myeloma in young and in select, fit, elderly patients. The procedure’s superiority was initially proven by the Intergroupe Francophone du Myeloma (IFM) and later confirmed by the UK Medical Research Council^5–8^. Despite its confirmed significant impact on event-free survival (EFS), multiple trials failed to depict significant impact on overall survival (OS)^9^. Even though standard, the procedure is still challenged by inevitable relapses threatening long-term remissions, and is therefore challenged by some myeloma experts who delay ASCT till relapse or progression^5,8^.

The introduction of drugs such as thalidomide, lenalidomide, and bortezomib administered before and/or after HDT/ASCT gave way to the groundbreaking achievement of stringent complete response (sCR) with a normal kappa/lambda ratio (serum free light chain)^10^, immunophenotypic CR^11^, and molecular CR^12^, in addition to significantly increased CR and CR plus very good partial response rate (VGPR)^13,14^. Thus, in the era of novel agents, embarking on transplantation is further scrutinized. Current ongoing studies are investigating incorporating different agents such as daratumumab and lenalidomide before and after transplantation, respectively. The challenge is thus to evaluate the necessity of HDT/ASCT when a powerful monoclonal antibody is combined with an induction regimen incorporating an immunomodulatory drug (IMiD) and a proteasome inhibitor (PI). This review addresses ten questions around the different steps of transplantation and lays forward current evidence from ongoing trials to aid decision-making.

## Which patients are candidates for ASCT?

There is no consensus regarding an age cutoff beyond which treatment with ASCT becomes questionable and as such, practice varies across institutions and countries. Generally, HDT/ASCT is reserved for patients younger than 65 years old with no severe comorbidities. Two important conditions are described below.

### Patient is over 65 years of age

Most of the randomized studies have included patients younger than 65 years of age and so it becomes difficult to infer conclusions regarding this matter^15^. Usually, age of participants is limited to 65 years to avoid selection bias and limit toxicities and withdrawal from studies. However, this does not mean that ASCT is not feasible in older patients. On the contrary, it is in select patients^16^. A previous study whereby the median age of patients was 72 years old concluded that elderly multiple myeloma patients should not be excluded from transplantation displaying good results with melphalan 140 mg/m^2^ (ref. ^16^). In the very few studies that did include older patients, melphalan doses were reduced (100 mg/m^2^ instead of 200 mg/m^2^) and the transplant procedure was repeated twice^5^. In a single center’s experience, “young” patients (age range 30–65) who received high-dose melphalan (HDM/ASCT (200 mg/m^2^)) and “elderly” patients (age range 66–75) who received two cycles of HDM/ASCT (100 mg/m^2^) were compared^17^. The analysis demonstrated no significant difference in progression-free survival (PFS), OS, or treatment-related mortality between the two groups and among all subgroups^17^. Interestingly, PFS and OS in “elderly” patients appeared to improve after 2008, due to the increased incorporation of novel agents in the treatment, thus leading to the conclusion that the combination of ASCT and novel-based regimens were not subject to the influence of age on treatment outcome^17^.

Currently, in the United States, fit patients up to 75 years old, receive ASCT^16^. On the other hand, in Europe, autologous transplants go up to the age of 70 off-protocol^18–20^. The field of transplantation among elderly patients still lags behind and awaits randomized controlled trials (RCTs) to synthesize solid guidelines.

### Patient has renal impairment

Renal impairment, per se, is not a contraindication to receiving HDT/ASCT. Nonetheless, it is a prompt reason to consider lower doses of therapy, as patients with renal impairment are more likely to suffer from HDM toxicities^21,22^. Studies, including the DAUTOS observational study of the Polish myeloma study group, demonstrated that dialysis-dependent patients were more likely to develop toxicities and complications such as mucositis and infections, but had PFS and OS comparable to matched patients with normal renal function^23,24^. Also, the dose of melphalan mattered, with patients achieving better outcomes with 200 mg/m^2^ (ref. ^24^). Interestingly, a proportion of patients were able to attain dialysis-independence after transplantation^24^. RCTs are yet to pave the way to guidelines regarding this transplantation scenario.

## What is the best induction treatment prior to ASCT?

The role of induction chemotherapy prior to HDT/ASCT is to decrease tumor burden, thus deepening the response rate and increasing the likelihood of engraftment, while retaining the maximum possible tolerability and minimum possible toxicity on normal hematopoietic cells. As a result, and prior to the introduction of novel agents, alkylating agents were avoided during induction, and regimens were dexamethasone-based such as the VAD regimen (vincristine, doxorubicin and dexamethasone)^1^. With the advent of new drugs, multiple trials have proven the superiority of induction regimens containing one or two novel agents (thalidomide or bortezomib) over the VAD regimen in increasing CR, CR plus near-complete response (nCR), or VGPR rates pre- and post ASCT^25–28^. Trials that compared two-drug (TD: thalidomide–dexamethasone or VD: bortezomib–dexamethasone) to three-drug induction (VTD: bortezomib, thalidomide, dexamethasone) have proven supremacy of the latter combination^13,29,30^. VTD was also proven superior to bortezomib, cyclophosphamide, and dexamethasone (VCD), thus highlighting the synergistic effect of combining an IMiD with bortezomib and dexamethasone^31^. As such, VTD became a standard induction regimen, whereby the role of a PI such as bortezomib is irreplaceable due to its demonstrated usefulness in high-risk patients^26,32,33^. Furthermore, although the general practice is to use 3–4 cycles of VTD before transplant, the use of 6 cycles of VTD was associated with deeper responses. This is to be weighed against increased side effects, specifically neuropathy, upon administering 6 cycles instead of 3–4 (ref. ^30^).

Similarly, the two-drug regimen, lenalidomide and dexamethasone (RD), was compared to bortezomib, lenalidomide, and dexamethasone (VRD) whereby VRD resulted in significantly increased PFS, response duration, and OS resulting in the IFM introducing VRD as induction^8,34,35^. In addition, the PETHEMA/GEM trial investigated induction with VRD-GEM with full dose lenalidomide from days 1 to 21, demonstrating an ORR of 85% post induction and 58% of patients achieving MRD-negativity post consolidation^36^.

Daratumumab (DARA), an anti-CD38+ monoclonal antibody, has been evaluated in patients with refractory disease^37,38^. The Cassiopeia phase III trial and the Griffin phase II trial compare DARA-VTD to VTD and DARA-VRD to VRD, respectively, demonstrating hopeful results of adding daratumumab^39–41^. Daratumumab plus cyclophosphamide, bortezomib, and dexamethasone (Dara-CyBorD) during induction was investigated in the phase II Lyra trial. Recent updates of the trial demonstrate activity and tolerability of Dara-CyBorD irrespective of high-risk cytogenetics with 12-month PFS and OS rates of 87% and 99%, respectively^42^. Finally, daratumumab is also being combined with carfilzomib, lenalidomide, and dexamethasone (KRD) in a phase Ib trial, whereby the combined regimen yielded 100% ORR, 91% ≥VGPR, and 43% ≥CR, with no negative impact on stem cell harvesting while retaining consistency of the DARA-KRD safety profile^43^.

MRD negativity, defined as the absence of disease within one million bone marrow cells, has been examined due to its important prognostic value at different stages of the transplantation process. The depth of response after induction and before ASCT determines patients’ prognoses after ASCT since the quality of response post induction and prior to ASCT are predictive of long-term PFS post ASCT^13,33,44–49^. The final analysis of the IFM2009 prospective trial demonstrated the significance of MRD negativity, whereby patients achieving MRD negativity after induction with VRD had a similar OS irrespective of whether they received an ASCT or not^50^. In addition, MRD negativity proved to be a more powerful predictor of outcome than cytogenetics, whereby patients with high-risk cytogenetics who achieved MRD negativity had better outcomes than patients with standard-risk cytogenetics who did not^50^. This could mean that MRD could potentially become essential in stratifying patients during maintenance and consolidation randomization and when deciding on maintenance duration^50^.

**Fig. 1:**
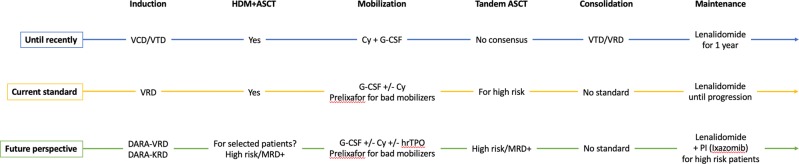
. Evolution of ASCT in multiple myeloma

With no evidence that four-drug regimens (IMiD, PI, alkylating agent, and steroid) are superior^51,52^, VTD and VRD remain the most currently used pre-transplant induction regimens, awaiting the results of ongoing trials testing the efficacy of adding daratumumab or the possible substitution of bortezomib with carfilzomib, which has been found to be safe and well tolerated with exceptional response rates (Fig. 1)^53,54^.

## What is the best stem cell mobilization procedure prior to ASCT?

A critical and essential step prior to ASCT is mobilization of hematopoietic stem cells (HSC) from the bone marrow to be harvested in peripheral blood. The minimum CD34+ stem cell dose considered sufficient for successful engraftment is 2 × 10^6^ cells CD34+/kg, but the optimal target is usually set at 5 × 10^6^ CD34+/kg. This can be done by steady-state mobilization with granulocyte colony-stimulating factors (G-CSF)^55^ or chemo-mobilization by the addition of chemotherapeutic agent(s)^5^. Currently, two G-CSF cytokines are approved for the mobilization of autologous HSC: filgrastim (10 µg/kg/day for 4–6 consecutive days and apheresis on days 5 or 6) and lenograstim (10 µg/kg/day for 4–6 days and apheresis between days 5 and 7)^5^. Even though tolerable, the use of G-CSF cytokines can yield suboptimal peripheral HSC harvest^56^. The most commonly used agent for chemo-mobilization is high-dose cyclophosphamide (2–4 g/m^2^)^57^, followed by filgrastim or lenograstim (5 µg/kg/day 1–5 days after completion of chemotherapy till last apheresis)^5^. This strategy can potentially decrease tumor burden, but the time to peripheral blood stem cell (PBSC) harvest is prolonged, with increased side effects^5^. Since some patients fail to mobilize^58–60^, the addition of new mobilization agents such as plerixafor, a chemokine-receptor 4 (CXCR4) antagonist, enhanced the stem cell mobilization effect of G-CSF^56,61^. Even though proven to be highly effective, plerixafor is not widely available^62^.

Other studies have compared cyclophosphamide to mCVAD (modified cyclophosphamide, vincristine, doxorubicin and dexamethasone) and mCBAD (modified cyclophosphamide, bortezomib, doxorubicin, and dexamethasone) concluding that more intense regimens are not superior to cyclophosphamide alone in mobilization^63^. A randomized phase III trial compared mobilization with cytarabine (Ara-C) and G-CSF versus G-CSF alone demonstrating improved yields with the addition of Ara-C, but increased hematologic toxicities^64^. It is noteworthy that employing lenalidomide in induction has been found to be one of the factors compromising stem cell mobilization success^65,66^, mainly due to lenalidomide upregulating CXCR4 and increasing the binding of stem cells to the stroma^67^. A prospective randomized phase II sub-study in the Finnish Myeloma Study Group-MM02 trial compared low-dose cyclophosphamide (2 mg/m^2^) plus G-CSF to G-CSF alone for mobilization in patients who have received lenalidomide during induction^68^. The addition of cyclophosphamide to G-CSF was superior, although G-CSF could yield similar results in patients receiving no more than three cycles of VRD^68^. In addition, the addition of plerixafor for mobilization in patients who received lenalidomide has also been proven effective^69^. As such, with the availability of plerixafor, the prolonged use of lenalidomide does not hinder stem cell mobilization. Finally, combining human recombinant thrombopoietin (hrTPO) to G-CSF and cyclophosphamide improved yields compared to cyclophosphamide and G-CSF alone^70^.

Therefore, the most currently used regimens are either chemo-mobilization with high-dose cyclophosphamide plus G-CSF or steady-state mobilization alone, with preemptive use of plerixafor when suboptimal mobilization is predicted by a low circulating CD34 count.

## What is the optimal conditioning regimen prior to ASCT?

The current accepted standard for HDT is intravenous high-dose melphalan (200 mg/m^2^). Previous trials attempting to replace this with oral and intravenous busulfan have failed, due to increased toxicity and lack of superiority, respectively^71,72^. The effect of intravenous busulfan is being studied in a phase III trial whereby HDM is compared to busulfan-melphalan (Bu-Mel: busulfan 130 mg/m^2^ daily for 4 days followed by two daily doses of melphalan at 70 mg/m^2^)^73^. The trial has demonstrated increased PFS with Bu-Mel without a significant difference in response rates^73^. Higher doses of melphalan (>200 mg/m^2^), which proved useful for patients with primary refractive or relapsing disease nearly 20 years ago^74^, are also being investigated. A randomized study comparing conditioning with melphalan 280 to 200 mg/m^2^ while receiving amifostine demonstrated significantly higher ORR and nCR without an improvement in OS and PFS in patients receiving melphalan 280 mg/m^2^ at the cost of higher incidence of grade 2–3 mucositis and gastrointestinal toxicities^75^. Another study demonstrated similar results with deeper responses on melphalan 280 mg/m^2^ without translating into improved survival^76^.

Bortezomib’s effect in transplant conditioning was investigated when combined with HDM in the IFM 2014-02 phase III study^77^. This showed no superiority of Bortezomib-HDM over HDM alone in terms of response rate, OS or PFS^77^. In addition, the role of bendamustine added to melphalan as part of conditioning is being explored, highlighting improved response rates and PFS^78,79^.

As such, HDM remains the standard conditioning regimen prior to ASCT awaiting results of clinical trials of other conditioning regimens (if any).

## What is the impact of consolidation therapy after ASCT?

The aim of short-term consolidation therapy after HDT/ASCT is to improve disease response with limited toxicity. Incorporating consolidation therapy in patients with a good response after ASCT was found to increase the CR rate and molecular remission, thus prolonging PFS^12^. The Italian Myeloma study group has previously investigated the effect of VTD versus TD as induction therapy before and as consolidation therapy after double ASCT, demonstrating VTD’s superior influence on CR/nCR rates and PFS^13^. Similarly, VRD proved superiority in consolidation^35^. These trials were very encouraging; nonetheless, randomized trials were needed to prove impact.

The second randomization in the EMN02/HO95 trial compared the aftermaths of receiving two cycles of VRD consolidation followed by lenalidomide versus lenalidomide maintenance alone, demonstrating the significant advantage VRD consolidation inferred in prolonging PFS^80^. Moreover, PFS was prolonged in most of the predefined groups in the study including ISS I and II, low-risk cytogenetics, irrespective of whether patients received VMP (bortezomib, melphalan, and prednisone) or transplantation prior to consolidation^80^. Nonetheless, VRD consolidation failed to improve PFS in patients with high-risk cytogenetics ((del17p and/or t(4;14) and/or t(14;16))^80^. This confirms the benefit of VRD consolidation followed by lenalidomide maintenance in younger, newly diagnosed multiple myeloma patients with low-risk disease^80^.

Along the same line of the EMN02/HO95 trial, the StaMINA phase III trial randomized patients to compare HDM/ASCT plus VRD consolidation plus lenalidomide maintenance, versus tandem HDM/ASCT plus lenalidomide maintenance, versus single HDM/ASCT plus lenalidomide maintenance^81^. It concluded that the addition of VRD consolidation or a tandem ASCT was not superior to standard ASCT followed by lenalidomide in upfront treatment of newly diagnosed multiple myeloma^81^.

With the currently available data, the role of post-transplant consolidation remains controversial.

## What is the impact of maintenance therapy after ASCT?

Even though HDT/ASCT is the standard frontline treatment for newly diagnosed multiple myeloma patients, ASCT is not curative, and progressions and relapses are common even if CR is attained post-transplant^6,7,82^. Maintenance therapy is thus added and is expected to be gentle with the safest profile post ASCT, but unlike consolidation, it is administered long-term to deepen the response, prevent progression, and prolong OS^5^.

Thalidomide, having already been used in different myeloma treatment settings and being an oral agent, has been tested in several randomized trials, most of which demonstrated benefit in terms of response rates but not OS^83–85^. Thalidomide was repeatedly associated with peripheral neuropathy, fatigue, and other side effects, all of which resulted in patient-reported decreased quality of life despite prolonged duration of disease control^85^. Thus, when used in the maintenance setting, the dosage and duration should be limited to 100 mg daily and 6–12 months, respectively, as suggested by Spencer et al.’s study^86^.

Lenalidomide maintenance has been shown to be well tolerated and to dramatically improve PFS and OS (Table 1)^87,88^. A recent meta-analysis of three RCTs, CALGB, IFM, and GIMEMA, that compared lenalidomide maintenance to placebo or observation, has demonstrated clinically valuable results^89^. Lenalidomide significantly improved PFS in all subgroups of patients regardless of age, myeloma severity and staging, and induction regimen (52.8 versus 23.5 months), even though patients who had received lenalidomide in induction, or had achieved a deeper response post-transplant, were more likely to benefit from lenalidomide^89^. OS was also significantly improved in the lenalidomide arm, except in women older than 60 years with poor cytogenetics^89^. Overall, the addition of lenalidomide reduced the chance of death by a substantial 25%, thus increasing median survival by approximately 2.4 years^89^. As demonstrated in previous studies, an increased incidence of second primary malignancies, albeit modest, was associated with lenalidomide, though the time to death due to a second primary malignancy did not differ between the two groups^89^. Such results propose lenalidomide as a standard maintenance drug in transplant-eligible patients^89^. Recent updates of the Myeloma XI trial’s results were in concordance with the meta-analysis^90^.

**Table 1:** Lenalidomide maintenance trials

Study	Median follow-up	*N*	Treatment	Outcome	
Meta-analysis	79.5 months			PFS	OS
IFM		605	Lenalidomide	52.8 months	Median OS not reached
CALGB		603	Placebo/Observation	23.5 months	86 months
GIMEMA				(HR 0.48; 95% CI 0.41–0.55)	(HR 0.75; 95% CI 0.63–0.9)
Myeloma XI	28.7 months			PFS	
		1136	Lenalidomide	60.3 months	
		834	Observation	30.1 months	
				(HR 0.47; 95% CI 0.39–0.57)	

So far in previous trials, lenalidomide has been given in low doses until progression or adverse events develop, and this practice is currently approved by both, FDA and EMA. Given that 30% of cases with premature termination of lenalidomide were attributed to toxicities and second primary malignancies^89^, the question that remains is regarding the optimal duration of treatment with lenalidomide for safety and cost.

Finally, bortezomib was also tested as part of maintenance, either alone or in combination with IMiDs, demonstrating improved PFS, but not OS^28^. Nonetheless, bortezomib poses an obstacle due to its subcutaneous/i.v. administration. The first oral PI, ixazomib, is currently being investigated. So far, it appears to have positive effects, with a safety profile comparable to that of lenalidomide alone, and is manageable by dose reductions (Fig. 1)^91^. Ixazomib was also compared to placebo in the multicenter TOURMALINE-MM3 trial with a median follow-up of 31 months, whereby there was a 39% improvement in PFS and a 28% reduction in progression or death. Ixazomib also allowed for deeper responses to be achieved^92^.

## What is the value of single versus tandem ASCT?

In the 1990s, in an attempt to improve survival and overall outcome, the concept of tandem transplant came about in an era where conventional chemotherapy was the only available drug^93^. Previous randomized trials had demonstrated improved outcomes with tandem transplantation in terms of PFS and OS even in patients who had not achieved a VGPR after the first transplant^1,94^. An alternative treatment approach, total therapy 3 (TT3), including induction, tandem ASCT, consolidation, and maintenance, has allowed one of the best results to be achieved (CR/nCR rate of 83%, 2-year PFS of 84%, and 2-year OS of 86%)^95^.

Long-term analysis of the GMMG-HD2 trial compared single versus tandem transplantation with conditioning with melphalan (200 mg/m^2^)^96^. The study proved the non-inferiority of single transplantation compared to tandem in the sense that OS and EFS did not significantly differ^96^. Nonetheless, the CR rates were significantly improved after the second transplantation^96^. Due to high drop-out rates, lack of use of novel therapy, and lack of subgroup analysis, the results of this study are to be cautiously interpreted^96^.

In the era of novel drugs, we needed trials to evaluate the impact of tandem transplantation such as the EMN02/HO95 and StaMINA trials^81,97^. The EMN02/HO95 trial explored the result of tandem versus single transplantation in newly diagnosed multiple myeloma patients^97^. Tandem transplantation was shown to improve the depth of the response by 25% with more than 50% of the patients achieving at least a CR^97^. PFS and OS were significantly improved after a second transplant, with approximately 30% reduction in the risk of death and progression^97^. Updated results of the EMN02/HO95 confirmed the improved 3-year PFS from 63 months after one ASCT to 73.1 after two ASCTs^98^. Importantly, the positive effect of tandem ASCT was seen in high-risk groups, in which randomization to receive double ASCT was found to be an independent predictor of PFS^97,98^. The analysis thus concluded that double frontline ASCT was superior to single ASCT in terms of PFS and OS in all patients, including poor prognosis subgroups, indicating that the latter were the most likely to benefit^97,98^.

On the other hand, the StaMINA trial failed to show superiority of tandem versus single transplant in the era of novel agents^81^. It is noteworthy that more than 30% of patients randomized to tandem transplant did not receive the second transplant^81^.

Overall, with the currently available data, a second ASCT may be beneficial in high-risk patients including patients with high-risk cytogenetics and RISS 3 category of disease.

## What is the added value of HDT/ASCT in the era of triple novel agent regimens?

With the advent of novel agents, it becomes questionable whether or not HDT/ASCT has any added value at all. The previously mentioned SWOG S0777 trial compared outcomes of lenalidomide and dexamethasone alone (RD) to bortezomib, lenalidomide, and dexamethasone (VRD) without an intent to transplant^34^. The results confirmed the superiority of VRD in increasing PFS, response duration, and OS^34^. As such, it is suggested that VRD alone is not only safe but has comparable PFS/OS to HDT/ASCT. Trials were thus necessary to compare novel agents in combination to ASCT to novel agents alone.

A randomized phase III trial for the IFM2009 was conducted to compare the efficacy of combination therapy with lenalidomide, bortezomib, and dexamethasone (RVD) alone to RVD plus HDT/ASCT in newly diagnosed multiple myeloma patients younger than 65 years old^8^. Patients were randomized so as to receive induction therapy with three cycles of RVD, and then consolidation with either five more cycles of RVD or high-dose melphalan followed by ASCT and two cycles of RVD^8^. All patients received lenalidomide maintenance for 1 year^8^. The use of transplantation in addition to novel agents as opposed to RVD alone resulted in significant improvement in PFS (50 versus 36 months, adjusted HR 0.65), CR rate (59% versus 48%), MRD negativity (79% versus 65%), and median time to disease progression (50 versus 36 months), with no advantage regarding OS^8^. In the phase III EMN02/HO95 study mentioned earlier, the first randomization compared the outcomes of HDT/ASCT (single or double) versus bortezomib–melphalan–prednisone (VMP) after induction with VCD^97^. Even though bortezomib has been repeatedly shown to increase PFS and OS, upfront ASCT was associated with decreased risk of progression and death and improved 3-year PFS irrespective of initial prognostic factors^97^.

On the other hand, two studies whereby transplantation was compared to alkylating agent-based regimens and lenalidomide associated a survival benefit with first-line transplantation^99,100^. Nonetheless, these trials did not incorporate bortezomib in their non-transplant arm which could explain the improved OS^99,100^.

The extent of improved PFS in the transplant arm in both EMN02/HO95 and IFM2009 trials, likely attributed to a deeper response through increased CR and MRD rates, suggest that given more observational time, we could possibly find an improvement in OS as was the case for lenalidomide maintenance. This is especially relevant given that relapsed patients receive comparable treatment including a second ASCT and the use of newly introduced agents.

The next challenge is to evaluate the necessity of HDT/ASCT when a monoclonal antibody such as daratumumab is added to a powerful induction regimen combining an IMiD and a PI, and whether this strategy can cure a fraction of patients. As such, we conclude that ASCT remains first line even in the era of novel agents. The impending challenge remains whether or not transplantation will be later substituted by less intensive novel agent combinations.

## What is the value of early versus late ASCT?

Frontline HDT/ASCT has been the standard for treating newly diagnosed multiple myeloma in young, fit patients and select elderly patients. Nonetheless, with the advent of present novel therapies, specialists have challenged the notion that HDT/ASCT should be administered early after diagnosis.

In 1998 before the era of novel agents, Fermand et al.^101^ studied the effect of autologous transplantation timing (early versus late) on OS. Patients who were randomized into the “early” arm received HDT/ASCT right away and those in the “late” arm received conventional chemotherapy until progression or relapse whereby they were supported with HDT/ASCT as well^101^. There was no difference in OS between the two groups^101^. Time without symptoms, treatment and treatment toxicity (TWiST) was also evaluated whereby the period spent without chemotherapy was longer in patients who received early HDT/ASCT, suggesting a clinical benefit of early versus late transplantation^101^. Several retrospective trials failed to demonstrate benefits in OS when comparing early to late HDT/ASCT, which could be attributed to selection bias regarding patients in the “late” group^102^.

As previously mentioned for the IFM2009 trial, comparing VRD to VRD plus transplant yielded significantly better outcome with upfront ASCT in terms of CR rate, PFS, and MRD negativity^8^. This highlights that, even in light of novel agents which have already been proven to drastically improve treatment outcomes, transplantation could further improve results. Nonetheless, OS was not affected by ASCT taking into account that transplantation was only done in two-third of the cases due to age, progression, and comorbidities, indicating that the benefits of upfront ASCT can be weighed against the toxicities of chemotherapy and transplantation, especially since late transplantation could secure a similar OS to early transplantation^8^. As such, in the absence of improvement in OS, delayed ASCT could be an option.

In the phase III EMN02/HO95 study mentioned earlier, the first randomization compared the outcomes of HDT/ASCT (single or double) versus VMP after induction with VCD^97^. Even though bortezomib has been repeatedly shown to increase PFS and OS, upfront ASCT was associated with a 24% reduction in risk of progression or death^97^. The estimated 3-year PFS was also significantly higher with upfront ASCT, regardless of the presence of poor prognostic factors^97^.

As such, it is safe to conclude that ASCT can improve outcomes whether performed as first line or as a rescue treatment^1,101^. Therefore, frontline ASCT remains the standard of treatment for fit, young and select elderly patients with newly diagnosed multiple myeloma.

## What is the role of ASCT as salvage therapy?

Salvage therapy is defined as ASCT given to a patient with signs of disease progression after an earlier ASCT^103^. By the BSBMT/UKMF Myeloma X trial, salvage ASCT with 200 mg/m^2^ melphalan was superior to cyclophosphamide 400 mg/m^2^ weekly for 12 weeks upon relapse and re-induction with VAD^104^. The time to disease progression (19 versus 11 months) and OS (67 versus 52 months) were significantly in favor of salvage ASCT^104^. As such, ASCT can be considered for salvage in fit patients if the interval between the first ASCT and relapse is 18 months or more^5,105^. This awaits trials that compare salvage ASCT with novel agents including the German study by Goldschmidt et al. comparing salvage ASCT to lenalidomide/dexamethasone.

## Conclusion and future prospects

Up until today, 30 years after its introduction, HDT/ASCT remains the standard of care for patients with newly diagnosed multiple myeloma. Despite the advent of novel agents, ASCT remains a very common treatment modality, especially in Europe and is included in all ongoing and proposed trials. The latter is yet to be challenged by many novel agents (including earlier use of CAR T cells) which are continuously explored. As such, and by projecting the results of the ALCYONE trial which proved superiority of DARA-VMP over VMP in transplant-ineligible patients^106^, we conclude that there appears to be a place for daratumumab in future induction regimens, due to its promising preliminary additive effect to triple regimens in terms of response. Multiple trials are currently looking into daratumumab in transplant-ineligible patients with recently released updates, suggesting that the addition of daratumumab allows deeper response, increased PFS, and improved MRD negativity^107^. Such results could be possibly extrapolated to the subset of patients who are newly diagnosed and are transplant eligible. Since melphalan, a cornerstone in conditioning prior to ASCT, is usually given at a fixed dose, patients can either reach concentrations that are above the median, thus sustaining survival but suffering increased toxicity or they can fail to reach median concentrations and suffer from untreated disease. To overcome that, pharmacokinetic (PK)-directed melphalan dosing is looked into as a potential means to determine optimal dosing in individual patients. Whether novel melphalan formulations such as FDA-approved propylene glycol-free melphalan have increased efficacy and decreased toxicity compared to melphalan is to be investigated. One study failed to demonstrate superiority of propylene glycol-free melphalan over melphalan^108^. Whether or not to embark on early transplantation is yet to be investigated; although it has been made clear that ASCT is synergistic to triple novel agent-based therapy, it could be delayed when toxicities outweigh benefits. When dealing with unfavorable cytogenetics and poor prognostic factors, tandem transplantation appears to be an encouraging strategy. Whereas the use of post-transplant consolidation is controversial, lenalidomide maintenance prolongs PFS and OS and can be considered as a standard of care. One important endpoint to be measured in future studies is MRD negativity in standard or high-risk disease, since it is a significant predictor of PFS, OS, and potential cure in a fraction of patients^109^. The objective is thus to achieve MRD negativity (<10^−6^), a predictor of better outcome^110^. To possibly achieve MRD negativity at even lower cutoff, daratumumab will likely need to be added. Finally, even though first line for multiple myeloma treatment, the use of transplantation remains limited by accessibility and availability which differs across nations and is limited in developing countries.
